# Evaluation of a Multichannel Non-Contact ECG System and Signal Quality Algorithms for Sleep Apnea Detection and Monitoring

**DOI:** 10.3390/s18020577

**Published:** 2018-02-13

**Authors:** Ivan D. Castro, Carolina Varon, Tom Torfs, Sabine Van Huffel, Robert Puers, Chris Van Hoof

**Affiliations:** 1 KU Leuven, Deptartment of Electrical Engineering—ESAT, 3001 Leuven, Belgium; carolina.varon@esat.kuleuven.be (C.V.); sabine.vanhuffel@esat.kuleuven.be (S.V.H.); puers@esat.kuleuven.be (R.P.); chris.vanhoof@imec.be (C.V.H.); 2 IMEC Belgium, 3001 Leuven, Belgium; tom.torfs@imec.be

**Keywords:** capacitive ECG, ECG quality indicator, non-contact ECG, sleep apnea, sleep monitoring, unobtrusive monitoring

## Abstract

Sleep-related conditions require high-cost and low-comfort diagnosis at the hospital during one night or longer. To overcome this situation, this work aims to evaluate an unobtrusive monitoring technique for sleep apnea. This paper presents, for the first time, the evaluation of contactless capacitively-coupled electrocardiography (ccECG) signals for the extraction of sleep apnea features, together with a comparison of different signal quality indicators. A multichannel ccECG system is used to collect signals from 15 subjects in a sleep environment from different positions. Reference quality labels were assigned for every 30-s segment. Quality indicators were calculated, and their signal classification performance was evaluated. Features for the detection of sleep apnea were extracted from capacitive and reference signals. Sleep apnea features related to heart rate and heart rate variability achieved high similarity to the reference values, with *p*-values of 0.94 and 0.98, which is in line with the more than 95% beat-matching obtained. Features related to signal morphology presented lower similarity with the reference, although signal similarity metrics of correlation and coherence were relatively high. Quality-based automatic classification of the signals had a maximum accuracy of 91%. Best-performing quality indicators were based on template correlation and beat-detection. Results suggest that using unobtrusive cardiac signals for the automatic detection of sleep apnea can achieve similar performance as contact signals, and indicates clinical value of ccECG. Moreover, signal segments can automatically be classified by the proposed quality metrics as a pre-processing step. Including contactless respiration signals is likely to improve the performance and provide a complete unobtrusive cardiorespiratory monitoring solution; this is a promising alternative that will allow the screening of more patients with higher comfort, for a longer time, and at a reduced cost.

## 1. Introduction

Unobtrusive monitoring of physiological signals is a field of research that has seen an increased interest during the last decades, due to its potential to enable a patient-centered healthcare with lower costs, higher population screening and an increased patient/user comfort. Non-contact, capacitively-coupled electrocardiography (ccECG) [1,2,3] is one of the technologies suited for long-term physiological monitoring without affecting people’s daily life; potential applications range from heart rate (HR) and heart rate variability (HRV) monitoring, to the diagnosis and follow-up of conditions involving the cardiorespiratory system. Despite this, the challenge of its use in real-life scenarios remains, due to its high sensitivity to motion artefacts, which leads to a high variation in the quality of the acquired signals. Furthermore, the real clinical value of these signals for specific medical diagnoses and disease monitoring, is yet to be validated in order to be adopted as a standard practice for patient monitoring and diagnostic screening.

Through-clothing ccECG measurements have been demonstrated to have high similarity to medical-grade electrocardiographic (ECG) recordings when using strapped bands [2,4] and even when integrated in everyday life objects [5,6], with a correlation of up to 90% [6]. Nevertheless, this high-quality ccECG is only obtained during certain periods of the unobtrusive monitoring; therefore, it is essential to be able to discriminate high-quality signals that can be used for health screening from artefact-contaminated ccECG only usable for a limited analysis, and even signals not useful at all due to high noise content or saturation resulting from poor electrode-body coupling. As a consequence, signal quality indication (SQI) algorithms for ccECG aiming artefact identification and quality estimation have been developed in recent years. These include the use of auxiliary signals such as estimated electrode-tissue impedance [7] and pressure [8], combined with additional processing steps such as adaptive filtering [7], regression models [8] and Principal Component Analysis (PCA) [9], each one involving specific additional processing stages for the binary classification of regions affected by motion. In some approaches, features extracted from the signal itself are also used, such as amplitude characteristics of the QRST complex [9], Wavelet decomposition features [10] and spectrum-based signal-to-noise ratio (SNR) estimations [11,12], either in combination with the aforementioned auxiliary signals or on its own, for segment or channel selection.

Signal quality estimation of conventional contact ECG has been investigated more extensively than for ccECG. These ECG quality metrics include the use of features such as match between different beat detection algorithms [13,14], multi-lead signal comparison [13,14,15], higher order moments such as Kurtosis [13,14,16], amplitude characteristics [16,17,18], spectral characteristics [13,14,15,19], HR characteristics [15,20], QRST area [21], QRST morphology [20], electrode-tissue impedance (ETI), and motion [22]; in some cases combined with additional processing such as Kalman Filtering [13], multi-layer perceptron neural networks (MLP) [14], support vector machines (SVM) [14], Least-Mean-Squares (LMS) adaptive filters, and PCA [22]. The high amount of research on ECG SQIs compared to the one on ccECG SQIs demonstrates the need for additional efforts to further validate these metrics in the contactless signals, due to the special characteristics of ccECG artefacts [23], which are not always the same as in their contact counterpart, and present a higher challenge.

There exist over 80 different sleep disorders [24], and it is estimated that up to 40% of the US adult population have problems falling asleep, mainly caused by disturbed sleep patterns [25]. The gold standard for sleep disorder diagnosis is a polysomnography (PSG) study: a high-cost and low-comfort test commonly performed during a one night stay at a hospital under the supervision of a clinician, in which multiple physiological signals are recorded, including ECG and respiration. Due to these costs and the comfort limitations, different approaches have been studied aiming to offer a more comfortable and lower cost alternative, ranging from the use of smartphones and bracelets to the home-monitoring of the complete set of PSG signals. For a detailed description of these developments the reader is referred to [26].

Given that ECG is one of the signals monitored in PSG, and due to the feasibility of home-based one-lead ECG monitoring, algorithms for the detection and monitoring of sleep disorders solely based on the ECG have been proposed [27,28,29]. There is a special interest in sleep apnea due to its high prevalence [30] and risk associated with a long-term effect on the cardiovascular system [31]. In an effort to further increase patient comfort and allow for a long-term sleep monitoring without affecting the person’s daily life, ccECG has been identified as a promising alternative to obtain ECG signals during sleep in an unobtrusive manner. ccECG from a bed has been measured by several researchers [32,33,34], but only a few of them have extracted features for sleep analysis [35,36]; to the best of our knowledge, none have shown a study towards sleep apnea analysis using ccECG signals, although it was mentioned as a possibility for further exploration by Takano et al. [34].

Considering that a non-contact sleep apnea monitoring solution would allow screening a higher number of patients with an added value of increased comfort, longer monitoring times, and less cost, this study presents, for the first time, the evaluation of a multi-channel ccECG system for the extraction of ECG features previously demonstrated to be highly effective in the automatic detection of sleep apnea [28]. Furthermore, an evaluation of multiple SQIs for channel selection, binary classification of signals into usable and not usable, and three-class classification of signals into usable, limited usability and not usable is presented. In addition, the obtained ccECG signals and extracted tachograms are evaluated against reference contact ECG signals.

## 2. Materials and Methods

### 2.1. ccECG Multichannel Acquisition

**Figure 1:**
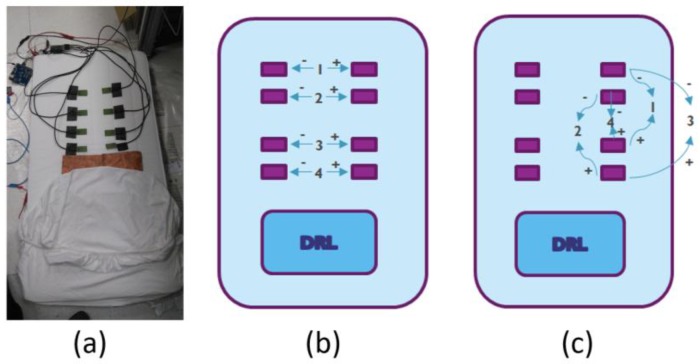
Mattress implementation of the multi-channel ccECG acquisition system and configuration for the different subject positions in the mattress. In (**a**) the system integrated in a mattress, before being covered with a cotton bed sheet; in (**b**) the electrode configuration for the four channels when recording signals from subjects lying on their back; in (**c**) electrode configuration for the four channels when recording signals from subjects lying on their left side; configuration for subjects lying on their right side is a vertically mirrored version of (**c**).

The research platform for the simultaneous acquisition of four ccECG channels presented in [6] was integrated on a mattress as shown in Figure 1a, and then covered by one layer of cotton bed linen. After signing an informed consent, volunteers were asked to place Ag/AgCl electrodes on each shoulder and their right ankle for reference Lead I ECG monitoring, and a strapped belt for reference respiration monitoring; both being acquired by a NeXus-10 MKII^®^ system (Mind Media, Herten, The Netherlands). The participants included six female volunteers and nine male volunteers, with ages between 20 and 43 years, and weight between 46 kg and 107 kg, all of them without any previously diagnosed cardiovascular disease. Each volunteer was then asked to lie on the mattress on his/her back during 10 min, on his/her right side during 10 min, and on his/her left side during 10 min, for a total recording of 30 min per subject. Differential electrode pairs for the four channels were configured as shown in Figure 1b for the recordings from the back, and as shown in Figure 1c for the recordings from the left side; recordings from the right side had the same configuration as Figure 1c but in the opposite electrode column. Following the three 10-min recordings, the volunteer was asked to place two additional Ag/AgCl electrodes in his/her back (in the middle-back, as a representation of the equivalent location for the ccECG electrodes) and to lie relaxed on the mattress, while 1-min simultaneous contact ECG signals were acquired from the front and the back, to evaluate the ECG differences explained by the location of the electrodes and not by the contactless technology when doing a ccECG vs ECG comparison. All the recorded data was encoded by a randomly generated 6-digit number, to guarantee the anonymity of the information.

### 2.2. ccECG and ECG Preprocessing and Alignment

Signals were obtained at sampling frequencies equal to 512 Hz, 256 Hz and 32 Hz for ccECG, ECG and respiration, respectively. All collected ccECG and ECG signals were digitally pre-processed as follows: high-pass filtered at a cut-off frequency of 0.5 Hz using a 4th order Butterworth filter, low-pass filtered at a cut-off frequency of 40 Hz using a 6th order Butterworth filter, and an additional Notch filter at 50 Hz using a 6th order Butterworth filter to remove any possible remaining interference from the power grid. All filtering operations were zero-phased by being applied in the forward and reverse directions, to avoid any shifts of the R peaks. After filtering, signal alignment was performed between ccECG and reference signals for all recordings.

### 2.3. Reference Signal Quality Labels

**Figure 2:**
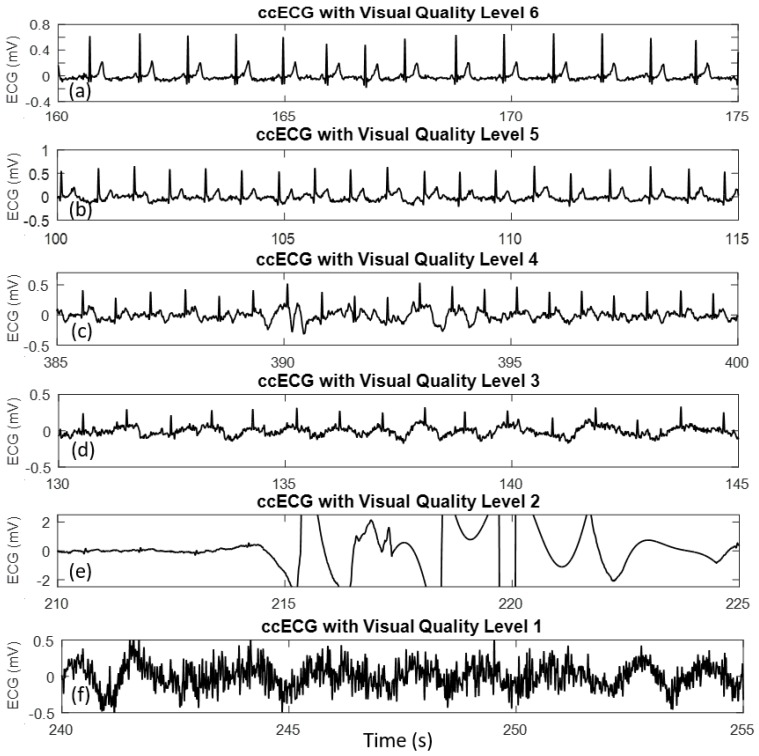
Example of ccECG signals acquired from mattress setup with different visually identified quality levels VQ. In (**a**) signal with VQ Level 6; in (**b**) signal with VQ Level 5; in (**c**) signal with VQ Level 4; in (**d**) signal with VQ Level 3; in (**e**) signal with VQ Level 2; in (**f**) signal with VQ Level 1.

A ground truth quality metric of the ccECG signals was required for: (1) Comparison against reference ECG; (2) Identification of the overall quality obtained; (3) Evaluation of the SQIs’ performance; (4) Classification of the extracted features for sleep apnea detection. In this context, ccECG signals were divided in 30-s segments, which is the typical signal segmentation in sleep monitoring techniques [37]; each segment was visually assessed and classified in 6 different Visual Quality (VQ) levels, where a higher number means a better signal quality. A detailed description of each of the quality levels and an example of a ccECG signal with each label is shown in Figure 2. Quantification of the segments in each quality group for the best channel per subject and best segment per subject were obtained as an indicator of the degree of usefulness of ccECG signals in a sleep environment.

In Figure 2, the Quality Level descriptions used as guidelines for the classification are defined as follows:

Level 6: Clean ECG looking like a standard contact Lead I ECG, full QRST morphology identifiable and relatively steady line between beats.

Level 5: Clear ECG detected, good QRST morphology. Few, almost no artefacts and noise. Morphology may be altered due to positioning, but signal is still clean. Beat-to-beat HR and HRV can be extracted, and morphology analysis is possible. When compared to a Lead I ECG morphology may differ in some cases due to configuration corresponding to a different ECG projection (Lead II or Lead III).

Level 4: ECG morphology identifiable and acceptable, including QRST. Some noise and artefacts are present, but in a small quantity. Beat-to-beat HR and HRV can be extracted, as well as some limited morphology analysis that may be affected by the artefacts present in the signal.

Level 3: R peaks in ECG identifiable, but QRST morphology less clear to identify, or artefacts are present in considerable amount, although peak detection may be recovered from it; or morphology significantly altered due to ballistocardiography (BCG)—which corresponds to the mechanical activity of the heart—or body positioning. Signal can be used for windowed HR extraction, and even for beat-to-beat HR extraction, although it may be corrupted in some cases.

Level 2: Some R peaks may be detected, but high amount of noise and/or artefacts are present that make the signal unusable, or intermittent ECG with some segments saturated or with big artefacts. This signal is not usable, as even a windowed HR analysis would result in distorted information.

Level 1: No usable ECG at all.

### 2.4. ccECG Comparison against Reference ECG

Non-contact signals were compared against reference ECG. For this, the correlation and magnitude squared coherence in the [0.67–40] Hz range (in accordance with IEC-60601-2-47) between ccECG and reference ECG were obtained for each best 30-s segment per measurement (obtained from the best channel and based on the VQ labels). In addition, beat detection statistics of ccECG with respect to the contact ECG were obtained for all the segments in each best-channel per measurement (based on averaged channel VQ labels), including Sensitivity and Positive Predictive Value (PPV) for beat matching, where a beat within ±150 ms is considered as a match [38]; correlation of the tachogram was also obtained as an additional comparison metric. Sensitivity and PPV were calculated as in (1) and (2) respectively, while Specificity and Accuracy were not calculated, since they require “Negatives”, “True Negatives”, and “False Negatives” that cannot be obtained when evaluating beat matching:

(1) $$Sensitivity=\frac{TP}{P}$$

(2) $$PPV=\frac{TP}{TP+FP},$$

where *TP* is the number of true positive beat matches, *P* is the number of ground truth beats and *FP* are the false detected peaks.

### 2.5. Signal Quality Metrics

Due to the importance of automatically identifying high-quality ccECG signal segments for a complete unobtrusive monitoring solution using capacitive sensors, several potentially useful SQI metrics were calculated for each of the 30-s segments collected, including spectral-based SQIs, a second-order moment SQI, a beat detector based SQI, and a morphology/template based SQI, as defined below:

**Spectral Density Ratio (SDR):** The spectrum-based SQI was calculated as the ratio of the power spectral density (PSD) of the signal in a narrow range of interest for ECG and a broader range that includes the characteristic frequencies of motion artefacts. In [13], Li et al. used a ratio between [5–14] Hz and [5–50] Hz for evaluation of ECG signals. Since ccECG motion artefacts have different characteristics than ECG artefacts, additional ranges were evaluated as an initial experimentation, obtaining 6 different SDR metrics calculated as in (3) with the limits shown in Table 1, where SDR1 corresponds to the metric calculated with the limits defined by Li et al.

(3) $$SDR=\frac{\int_{a}^{b}PSD}{\int_{c}^{d}PSD}$$

**Kurtosis (KUR):** It has been identified previously that the fourth order moment of the ECG signal (Kurtosis) can be used as an indicator of a clean or contaminated ECG. In [13] a Kurtosis-based SQI was used with a threshold of 5, following He et al. [39], who defined that a clean sinus rhythm ECG generally has a Kurtosis larger than 5. Gholinezhadasnefestani et al. [16] evaluated Kurtosis for the improvement of HRV in newborns, finding it performed rather poor when using it as a standalone continuous metric compared to standard deviation and RMS value of the signal, without reporting any optimized threshold. On the contrary, Clifford et al. [14] found Kurtosis as providing one of the best results when applying an SVM classifier to different individual metrics. With the aim to evaluate the KUR SQI metric for ccECG signals, the Kurtosis of each 30-s segment was calculated as in (4):

(4) $$Kurtosis=\frac{\frac{1}{n}\sum_{j=1}^{n}{\left(x_{j}-\tilde{x}\right)}^{4}}{{\left(\frac{1}{n}\sum_{j=1}^{n}{\left(x_{j}-\tilde{x}\right)}^{2}\right)}^{2}},$$

where *x* is the input signal, $\tilde{x}$ is mean value, and *n* the number of samples for each segment.

**Kurtosis Modified (KURMod):** In our previous experimental results with ccECG signals and calculations of Kurtosis, it has been observed that not only Kurtosis values lower than 5 are likely to be contaminated by artefacts, but that too high values may also indicate a corrupted ccECG. Following this, we propose a modified Kurtosis metric in which any value higher than or equal to 18 is replaced by 1, and therefore considered as a contaminated segment. This metric was also obtained for all the 30-s segments recorded.

**Beat detection-based indicator (bSQI):** The bSQI metric followed the method described in [13,14] for contact ECG, where two beat detection algorithms (Hamilton and Tompkins and Zong et al.) are simultaneously used, and the SQI is calculated based on the beat matching of both methods as shown in (5):

(5) $$bSQI=\frac{N_{m}}{N_{HT}+N_{Z}-N_{m}},$$

where $N_{m}$ represents the beats that are matched between both beat detection methods (within a range of ±150 ms [38]), $N_{HT}$ is the number of beats found by the algorithm by Hamilton and Tompkins, $N_{Z}$ is the number of beats found by the algorithm by Zong et al., and bSQI is a number from 0 to 1 indicating the quality of the ccECG signal. This indicator is based on the different sensitivity of both methods to artefacts in the signal, and was also obtained for each 30-s segment.

**Figure 3:**
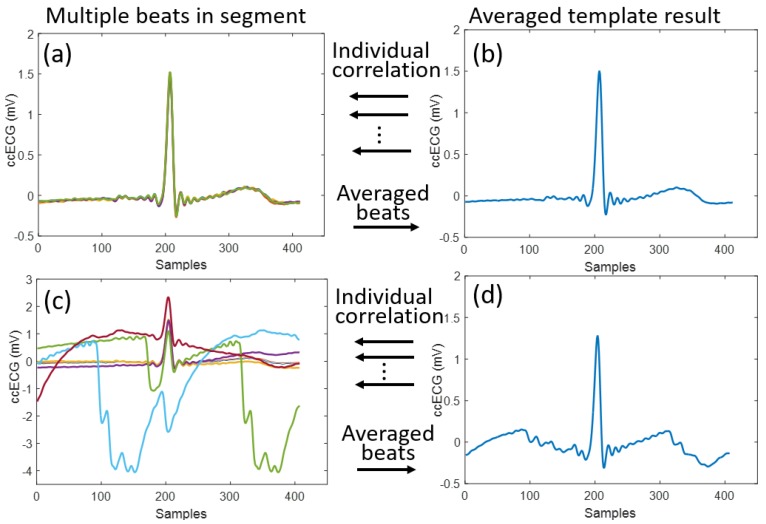
Example of template extraction for obtaining the morphology-based quality indicator denominated as corrSQI. In (**a**) a set of extracted beats from a clean ccECG segment is shown; these beats are then averaged to obtain the template shown in (**b**), and corrSQI is obtained by correlating this template to each individual beat in (**a**), leading to a high-quality metric. Likewise, (**c**,**d**) show the same procedure for a segment containing motion artefacts. Despite the artefacts, the template in (**d**) still has an ECG-like waveform, and can be used for the individual correlation with the beats in (**c**), resulting in a lower corrSQI than the one obtained from (**a**).

**Template correlation SQI (corrSQI):** The characteristic QRST morphology is one of the main features that can be useful to differentiate a higher-quality ECG signal from artefact-corrupted ECG. Despite this, it is difficult to have a universal, time-independent, person-independent template to compare against. Averaging of QRST complexes has been implemented in magnetocardiogram signals [40] with the aim of improving SNR, and template matching in ECG signals was used for diagnostic purposes [41,42]. Furthermore, a contact ECG and photoplethysmography (PPG) quality estimation based on template matching was recently proposed by Orphanidou et al. [20]. In this context, a similar procedure was applied to the ccECG signals in order to obtain a template-based SQI. For each 30-s segment, all the detected QRST complexes were extracted by using windows around the automatically detected [43]. R peaks as big as the average R-R period from the correspondent segment. Following this, the extracted beat windows were averaged, and this result was then used as a template to be correlated to the individual beats in the segment as shown in Figure 3, to obtain a beat-wise quality metric. These metrics were averaged for all the beats in the segment, resulting in the assigned segment corrSQI metric.

### 2.6. SQI-Based Signal Classification

Using the SQIs described in the previous section, quality-based segment classification was performed and evaluated. Two types of classification were done: the first one aimed to identify the usable ccECG segments from the not-usable ccECG segments by applying a threshold to each of the calculated SQI metrics; while the second one used the same metrics to divide the segments in three different classes: usable, limited usability, and not-usable, using two threshold values. We will name these binary and three-class classifications respectively. For the classification evaluation, all the collected segments were divided in the ones obtained from the back and from the side (including right and left). Furthermore, in each category the segments were divided in non-overlapping training and test sets, with a 70:30 distribution.

The optimized threshold for the binary classification was found by obtaining a Receiver Operating Characteristics (ROC) curve from each SQI for the signals in the training set (divided in back, side and all), and finding the cost-effective point in the ROC curve. Both the ROC and cost-effective point calculation were obtained by using the ‘ROCOut’ Matlab^®^ implementation of Cardillo [44], publicly available in the web. Following this, the test set was used to obtain the performance of the classification with the same method, but obtaining the metrics at the previously optimized threshold to avoid overfitting. The thresholds for the three-class classification were obtained by applying a Linear Discriminant Analysis (LDA) to the training sets and evaluated in the test sets.

The performance evaluation of the three-class classification was done considering VQs of 1 and 2 as not-usable signals, VQs of 3 as signals with limited usability, and VQs of 4 to 6 as usable signals. In the case of the binary classification, two different evaluations were done; one that included VQs of level 3 or higher as usable and one that included VQs of level 3 or lower as not-usable.

### 2.7. SQI-Based Channel Selection

Since the proposed system has more than two electrodes and allows for the simultaneous acquisition of four ccECG channels, there is also an additional need to select a best-quality channel out of the available sources. This is done with the aim of transmitting only the minimum necessary information and avoid running diagnostic algorithms in redundant or corrupted data sources. With this purpose, the performance of each of the SQI metrics presented in Section E was evaluated regarding the selection of the best channel for every set of 4 simultaneously recorded 30-s segments. For each SQI, the channel with the highest value was selected provided its value was higher than a base threshold of 0.74, 0.15, 0.52, and 4.2 for bSQI, SDR2, corrSQI and KURmod respectively. These thresholds were defined as 90% of the threshold values optimized for the binary classification using level 3 as threshold, including both back and side signals.

Likewise, the channel selection performance of a proposed “SQI-Fusion” algorithm that involves some of the SQI metrics, together with additional signal features was also evaluated; this selection was performed for each window of 4 simultaneously acquired 30-s segments. The algorithm involved three steps: (i) An SQI-based pre-selection of possible best-channels using: bSQI as in (6), SDR2 as in (7), corrSQI as in (8), and KURmod as in (9), where the thresholds were defined as indicated in the paragraph above; (ii) In a second step, the conditions of Table 2 were used to narrow the selection; (iii) A unified SQI (uSQI) as defined in (10) was used to select the best channel from the remaining potential candidates.

(6) $$sel_{bsqi}=\{\begin{array}{c} \mathrm{ccECG}\;\mathrm{with}\;\mathrm{highest}\;\mathrm{bSQI}:\\ if\;bSQI_{1st}\geq 0.74\\ 2\;\mathrm{ccECGs}\;\mathrm{with}\;\mathrm{highest}\;\mathrm{bSQI}:\\ if\;bSQI_{1st}\geq 0.74,\\ and\;bSQI_{2nd}\geq 0.74,\\ and\;bSQI_{2nd}\geq bSQI_{1st}*0.9\\ \mathrm{No}\;\mathrm{ccECG}\;\mathrm{selection}:\\ if\;bSQI_{1st}<0.74 \end{array}\;bSQI_{1st}:bSQI\;value\;of\;channel\;with\;highest\;bSQI\;bSQI_{2nd}:bSQI\;value\;of\;channel\;with\;2nd\;highest\;bSQI\;sel_{bsqi}:Set\;of\;selected\;channels\;by\;bSQI$$

(7) $$sel_{sdr2}=\{\begin{array}{c} \mathrm{ccECG}\;\mathrm{with}\;\mathrm{highest}\;\mathrm{SDR}2:\\ if\;SDR2_{1st}\geq 0.15\\ 2\;\mathrm{ccECGs}\;\mathrm{with}\;\mathrm{highest}\;\mathrm{SDR}2:\\ if\;SDR2_{1st}\geq 0.15,\\ and\;SDR2_{2nd}\geq 0.15,\\ and\;SDR2_{2nd}\geq SDR2_{1st}*0.9\\ \mathrm{No}\;\mathrm{ccECG}\;\mathrm{selection}:\\ if\;SDR2_{1st}<0.15 \end{array}\;SDR2_{1st}:SDR2\;value\;of\;channel\;with\;highest\;SDR2\;SDR2_{2nd}:SDR2\;value\;of\;channel\;with\;2nd\;highest\;SDR2\;sel_{sdr2}:Set\;of\;selected\;channels\;by\;SDR2$$

(8) $$sel_{corr}=\{\begin{array}{c} \mathrm{ccECG}\;\mathrm{with}\;\mathrm{highest}\;\mathrm{corrSQI}:\\ if\;corrSQI_{1st}\geq 0.52\\ 2\;\mathrm{ccECGs}\;\mathrm{with}\;\mathrm{highest}\;\mathrm{corrSQI}:\\ if\;corrSQI_{1st}\geq 0.52,\\ and\;corrSQI_{2nd}\geq 0.52,\\ and\;corrSQI_{2nd}\geq corrSQI_{1st}*0.9\\ \mathrm{No}\;\mathrm{ccECG}\;\mathrm{selection}:\\ if\;corrSQI_{1st}<0.52 \end{array}\;corrSQI_{1st}:corrSQI\;of\;channel\;with\;highest\;corrSQI\;corrSQI_{2nd}:corrSQI\;of\;channel\;with\;2nd\;highest\;corrSQI\;sel_{corr}:Set\;of\;selected\;channels\;by\;corrSQI$$

(9) $$sel_{kurm}=\{\begin{array}{c} \mathrm{ccECG}\;\mathrm{with}\;\mathrm{highest}\;\mathrm{KURmod}:\\ if\;KURmod_{1st}\geq 4.2\\ 2\;\mathrm{ccECGs}\;\mathrm{with}\;\mathrm{highest}\;\mathrm{KURmod}:\\ if\;KURmod_{1st}\geq 4.2,\\ and\;KURmod_{2nd}\geq 4.2,\\ and\;KURmod_{2nd}\geq KURmod_{1st}*0.9\\ \mathrm{No}\;\mathrm{ccECG}\;\mathrm{selection}:\\ if\;KURmod_{1st}<4.2 \end{array}\;KURmod_{1st}:KURmod\;of\;channel\;with\;highest\;KURmod\;KURmod_{2nd}:KURmod\;of\;channel\;with\;2nd\;highest\;KURmod\;sel_{kurm}:Set\;of\;selected\;channels\;by\;KURmod$$

(10) uSQI=SDR2∗bSQI∗corrSQI∗chvotes,

where *chvotes* corresponds to the number of “votes” that a channel received from the different SQIs.

**Figure 4:**
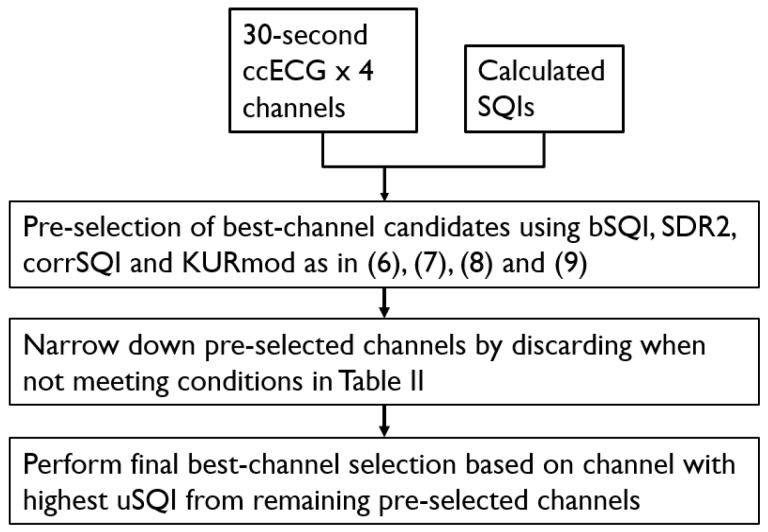
Block diagram of algorithm using SQIs and additional signal parameters.

A general block diagram of the algorithm is shown in Figure 4.

The selection of using KURmod instead of KUR allows to use it as a continuous metric with a defined threshold, while the use of SDR2 from the available SDR metrics was a result of an evaluation of the SDR that provided best performance for the fusion selection method.

### 2.8. Extraction of ccECG Features for Sleep Apnea Detection

With the purpose of evaluating the usefulness of ccECG signals acquired from a sleep environment in the extraction of features used for detection of sleep apnea, the features presented in [28], and reported to be effective in the automatic detection of this condition, were calculated for all the segments in every best-channel from each measurement, as well as for the correspondent reference ECG signals. These features comprise: (1) Standard Deviation (SD) of R-R intervals; (2) Serial correlation coefficient of the R-R intervals at a lag of *k* = 5 samples (CorrCoeff); (3) A PCA feature obtained from the stacked QRST complexes in the segment (PCAF); (4) The SD of the ECG-derived respiration (EDR) estimated based on the R peak amplitude method; (5) The relative power of the HR signal component explained by the respiratory activity (RelPowResp); (6) The relative power of the HR signal component not explained by the respiratory activity (RelPowResi). For detailed information regarding these features the reader is referred to [28].

## 3. Results

After data collection from the 15 volunteers in the three defined positions (Back, Right, Left) during 10 min each (four simultaneous channels), a total of 3144 30-s ccECG segments were obtained, from which 1196 were recorded from the back and 1948 from the side (including right and left). The reason the total number of segments is not 3600 (equivalent to complete 30 min recording per subject) is because in some recordings the last segment was less than 30 s, and 4 of the subjects did not obtain a right-side recording due to limitations in the alignment with the electrodes; despite this, the statistics of the ccECG signal quality do include these 4 subjects with an assigned VQ of 1, as indicated in the following section. From the total number of segments obtained, 96 (24 4-channel sets) presented difficulties in aligning with its reference due to the different morphology of the signals acquired from the side, with respect to the reference Lead I, and were excluded from the analyses that involved the use of the reference ECG, leaving 3048 segments (762 4-channel sets) available for this purpose.

### 3.1. ccECG Signal Quality

As a result of the visual quality levels VQ assigned to each segment, and in order to evaluate the overall quality of ccECG across subjects, Table 3 is presented with the quality distribution obtained when including the best segment from each measurement. If we evaluate the percentage of signals with a quality VQ level of 3 or higher, it is seen that this corresponds to 100% of the selected segments from the back, and to 83.33% of the segments from the side. As additional information, when including the complete acquired ccECG signal for each measurement (the best channel out of the four simultaneous) these percentages are only reduced to 97.99% and 77.60% respectively. The presented data includes the 4 subjects where a right-side recording was not possible and assigns a VQ of 1 for the not recorded signals, resulting in the 13.33% of VQ 1 for the side measurements shown in Table 3.

### 3.2. Comparison between ccECG and ECG Traces

**Figure 5:**
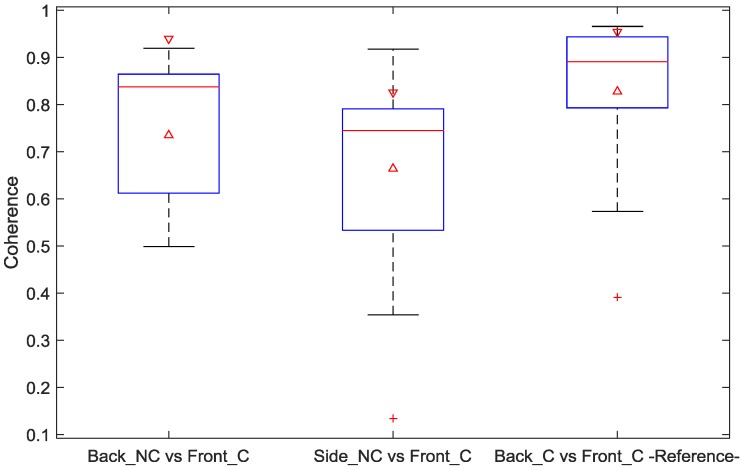
Coherence boxplot of signals between 0.67 Hz and 40 Hz. Left box shows the coherence between ccECG from the back and contact ECG from the front; middle box shows the coherence between ccECG from the side (left and right) and contact ECG from the front; right box shows the coherence between contact ECG from the back and contact ECG from the front for reference on the maximum similarity of signals obtained with the same reference method from these two locations. ccECG signals evaluated correspond to the collection of every best segment per measurement. NC: Non-Contact; C: Contact.

**Figure 6:**
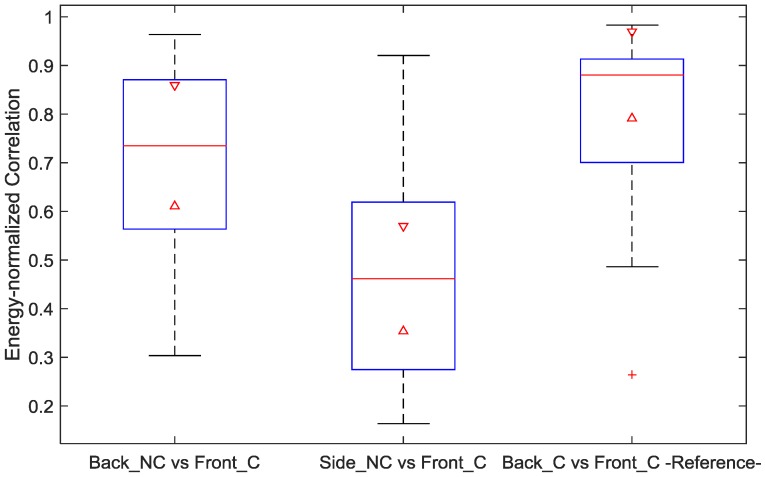
Correlation boxplot. Left box shows the correlation between ccECG from the back and contact ECG from the front; middle box shows the correlation between ccECG from the side (left and right) and contact ECG from the front; right box shows the correlation between contact ECG from the back and contact ECG from the front for reference on the maximum similarity of signals obtained with the same method from these two locations. ccECG signals evaluated correspond to the collection of every best segment per measurement. NC: Non-Contact; C: Contact.

The obtained signal coherence and correlation are shown in the boxplots of Figure 5 and Figure 6, respectively. The two left-most boxes show these metrics comparing ECG reference (from the front) with ccECG signals recorded from the back and from the side.

In addition, this comparison was also obtained for the 1-min ECG reference signals simultaneously obtained from the front and the back, as presented in the right-most box of each boxplot; this last calculation was intended to provide information regarding which part of the difference between reference ECG and ccECG signals is explained by the location of the electrodes and not by the use of the contactless technology. The results from Figure 5 show a high coherence between ccECG and reference ECG signals, with a median of 0.83 for the ccECG signals from the back and 0.74 for the ccECG signals from the side; maximum values of 0.91 were obtained in both cases. In addition, the reference ECG front-back coherence presents a median of 0.89 and a maximum of 0.96, which indicates that part of the difference between ccECG and reference ECG can be explained by the electrode location rather than the capacitive coupling of the electrodes. Likewise, Figure 6 presents correlations between ccECG and ECG with a median of 0.73 and 0.48 for back and side ccECG signals respectively, and maximum values of 0.96 and 0.92. The reduced correlation for signals from the side is a result of comparing two different ECG projections, namely Lead II with Lead I and Lead III with Lead I. The contact front-back correlations have a median of 0.88 and a maximum of 0.98, once again indicating that electrode positioning partly explains the difference between ccECG and ECG signals.

### 3.3. Beat Detection and Tachogram Comparison

Beat detection statistics from the complete 10-min recordings (each best channel per measurement) comparing ccECG and ECG are shown in Table 4 along with the averaged correlation of the tachograms, for further indication of the overall quality and usefulness of ccECG measurements compared to the clinical-grade ECG. It can be seen that when including all the positions, all the metrics are higher than 95%, and that this increases to more than 98% when only considering measurements from the back; measurements from the side present slightly lower metrics but still higher than 92%.

### 3.4. Binary and Three-Class Segment Classification

The results of the tests for the binary classification using the ROC-optimized thresholds are presented in Table 5 when considering a VQ level 3 as threshold for usable signal, and in Table 6 when considering a VQ level 4 as threshold for the same classification. These tables include the ROC-optimized threshold, as well as the Sensitivity, Specificity, PPV and Accuracy of the method for signals from the back, from the side, and a combination of all signals. A maximum classification accuracy of 91% was obtained when using the corrSQI indicator with a threshold of 0.43 for the signals recorded from the back and considering VQ level 3 as usable threshold (Table 5); for this same VQ threshold, corrSQI also performed better when applied to signals from the side and to the complete set of signals, with accuracies of 77% and 82% respectively. When considering VQ level 4 as usable threshold (Table 6), both corrSQI and bSQI presented higher performance than the other metrics, with accuracies between 86% and 87% for all the signal groups (back, side, and combined). It is worth noting that although maximum accuracy for signals from the back was obtained for VQ level 3 as threshold, the highest accuracies when including all the signals were obtained for VQ level 4 threshold with bSQI and corrSQI having similar performance (87% and 86% respectively).

When testing the classification of the segments in three different classes by using thresholds obtained by a LDA, lower performance was obtained for all the SQIs; corrSQI metric outperformed all the other metrics with an overall Accuracy of 79% for the signals from the back, and 70% when including both back and side position signals. The confusion matrix for this metric, for the set of all measurements, is presented in Table 7, where it is seen that the classification of the “Usable” and “Not Usable” classes kept a relatively high sensitivity of 87% and 82% respectively, while the “Limited Usability” class had a low Sensitivity of 31%.

### 3.5. Automatic Best-Channel Selection

Automatic best-channel selection by each of the presented SQIs was evaluated and compared against selection by the visual VQ level, obtaining the results presented in Table 8. Sensitivity and PPV were calculated for all the signal groups (back, side, and both combined); two scenarios were evaluated in each case: One that considered a channel selection match when the SQI-selected channel was the same as the highest VQ channel (resulting in Sens0 and PPV0), and one that allowed an error of 1 VQ level and also considered a match when the SQI selected channel was the one with the second-highest VQ, as long as it was VQ level 3 or higher (resulting in Sens1 and PPV1). Results are presented for each of the SQIs previously described, as well as for the “SQI-Fusion” algorithm and a second version of this fusion that does not involve corrSQI (SQI-Fusion *w*/*o* corrSQI), in order to evaluate if the fusion of the other methods outperformed this best-performing SQI. It can be seen that once more, corrSQI presents the highest performance, with a maximum Sensitivity of 94% and PPV of 92% when considering signals from the back and allowing an error of 1 VQ level. Likewise, in all the groups the metrics are consistent, indicating the best performance for corrSQI even above the fusion and the fusion without corrSQI. Although the performance of bSQI and KurtosisMod was lower, these SQIs still showed an acceptable capability of best-channel selection, with metric performances between 80% and 86% for signals from the back, and between 69% and 74% for all the signals, but with lower performance for signals from the side.

### 3.6. Extraction of Features for the Detection of Sleep Apnea

**Figure 7:**
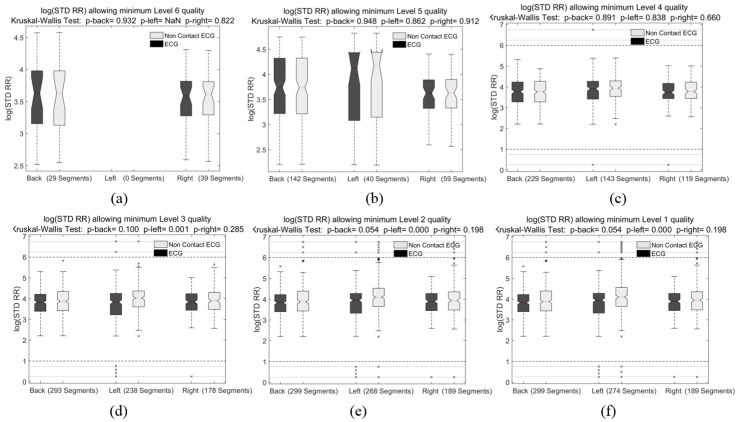
Results of extracting the standard deviation of the RR intervals from all the 30-s segments in the best channels per measurement. The extracted feature from ccECG and reference ECG are presented, together with Kruskal-Wallis Test *p*-values. The boxplots present the results of including the signal segments with a Visual Quality of Level (**a**) 6; (**b**) 5 and 6; (**c**) 4 to 6; (**d**) 3 to 6; (**e**) 2 to 6; (**f**) 1 to 6. Kruskal-Wallis Test results show that when including the signals of quality 3 and lower, the *p* value drops significantly.

**Figure 8:**
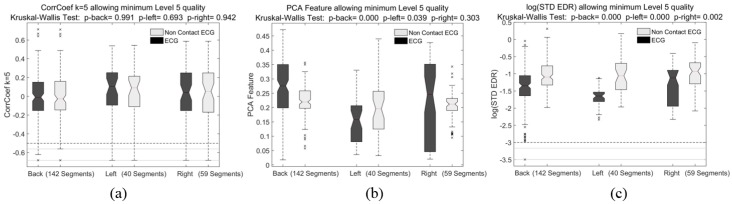
Results of extracting serial correlation coefficient of R-R intervals CorrCoeff (**a**); a PCA-based feature (**b**); and the SD of the EDR (**c**) from all the 30-second segments in the best channels per measurement. The extracted features from ccECG and reference ECG are presented, together with Kruskal-Wallis Test *p*-values. The boxplots present the results of including the signal segments with a Visual Quality of Level 5 and 6. Kruskal-Wallis Test results show that the CorrCoef feature is not significantly different between ccECG and ECG, but that the PCA-based feature and the SD of the EDR are significantly different between the two methods. The remaining features regarding the relative power and relative residual power of the HR component (not shown) were also significantly different when extracted from ccECG and ECG, since they include the PCA feature and EDR.

The results of extracted features for the detection of sleep apnea were grouped by minimum VQ quality level as shown in Figure 7 for the SD of R-R feature. The results for the features CorrCoeff, PCAF and SD of EDR for a minimum VQ level 5 are presented in Figure 8, and the obtained features RelPowResp and RelPowResi are not shown, as they presented a similar performance than PCAF and SD of EDR.

Figure 7 clearly shows that the quality of ccECG signals influences the accuracy of the feature extraction, and that signals with VQ level 4 or higher should be selected for this purpose. This is evidenced when evaluating the *p*-values of the Kruskal-Wallis tests, which are not higher than 0.3 when including signals with VQ level 3 (Figure 7d); on the other hand, this feature extracted for signals with minimum VQ of 4 resulted in *p*-values of 0.89, 0.83 and 0.66 for the back, left and right measurements respectively (Figure 7c), and increased to 0.94, 0.86 and 0.91 when leaving only level 5 and level 6 signals, as shown in Figure 7b.

Following this analysis, the results of minimum VQ 5 presented in Figure 8 show that the CorrCoeff feature is still significantly similar between ccECG and ECG signals with *p*-values of 0.99, 0.69 and 0.94, while the features PCAF and SD of EDR obtained from ccECG, differ significantly (*p*-values < 0.31) from that of the reference ECG, suggesting an effect of the contactless technology on these specific morphology-based features even for high-quality signals. Likewise, the two remaining (not shown) features related to the power and residual power of the HR with respect to the respiratory activity (RelPowResp & RelPowResi) also show low *p*-values even for signals with VQ 5 and VQ 6. This shows a clear distinction in which ccECG can accurately provide features related to HR and HRV, while the features derived from signal morphology present a greater challenge.

## 4. Discussion

The present study had the main objective of evaluating the use of ccECG signals for the extraction of features that allow the automatic detection of sleep apnea, as well as comparing the performance of different SQIs in the automatic signal classification and best-channel selection. In this regard, the presented results evidence that the features related to HR and HRV that are useful for sleep apnea analysis can be accurately extracted from ccECG signals, resulting in Kruskal-Wallis test *p*-values of up to 0.94 and 0.98 for the SD of R-R and CorrCoeff features respectively, when compared to the features extracted from the reference ECG; this is supported by the results that evaluate the beat detection and tachogram from ccECG, which show a beat-matching to the reference ECG of more than 95% for signals collected from the back and the side together, and more than 98% for signals collected from the back, with average tachogram correlations of about 97% and 99% respectively. Furthermore, it was clearly shown that to obtain these high similarity metrics for the sleep apnea features, it is crucial to only use high-quality signals, which in the framework of the defined VQs from Figure 2 correspond to signals with at least a VQ higher or equal to level 4.

Due to this requirement to perform a best-channel selection and a further segment quality classification, the presented SQI evaluation for segment classification and selection is an important step to assess a potential pre-processing solution to identify signals that can be used for the extraction of sleep apnea features. For quality classification, it was found that the corrSQI performed best among the evaluated metrics, with a classification accuracy of up to 91% when dividing “usable” from “not-usable” signals with a threshold of VQ level 3; nevertheless, since the extracted features presented best performance starting from a VQ level 4, it is more accurate to report the classification performance at this threshold level, which was higher for both corrSQI and bSQI metric with accuracies of 86% and 87% even when including signals from the back and from the side together, which indicates the potential usefulness of both SQIs as a pre-processing step. In addition, these two metrics presented a channel selection accuracy as high as 92% and 84% respectively, confirming their quality indication capability. In the case of three-class classification the obtained performances were lower (with a maximum overall accuracy of 79%), and it was clear that the “limited use” class may need a different way of classification, since it obtained a class sensitivity of only 31%. Despite this, it was shown that the binary classification is enough when aiming to extract accurate HR and HRV related sleep apnea features. It is worth to note that the thresholds optimized in this work for the data from healthy volunteers may vary for patients with sleep apnea; therefore, in future studies these thresholds should be verified by training on the patient data instead of healthy volunteers.

Although the comparison between ccECG and ECG traces resulted in relatively high coherence and correlation (except for the correlation of signals from the side), the morphology-related sleep apnea features, namely PCAF, SD of EDR, RelPowResp and RelPowResi obtained from the ccECG signals, presented a low similarity with the features extracted from the reference ECG signals even for high-quality segments. It is important to mention that these features quantify the mechanical effect that respiration has on the modulation of the ECG. They exploit the amplitude changes of the ECG that are caused by the relative movement of the electrodes with respect to the cardiac vector during a breathing cycle, and the change in chest impedance due to the filling of the lungs with air. Since these two effects are susceptible to be masked in ccECG by the variations in the electrode-tissue interface, differences in the morphology features are expected, as they may include other phenomena such as the change in capacitive coupling to the body. In addition, the acquisition of the signal from a different location may further contribute to the obtained difference when compared to the features resulting from the reference ECG. These results call for further analysis of these features in the ccECG case, which may need to be optimized as it was done in [28] for the contact ECG case; for instance, the second component PCAF feature was used as the one explaining the variance of the QRST complexes in relation to apneic episodes, but due to the modified morphology and additional movement-related artefact source, it could be possible that a different component is required.

The authors acknowledge that the method selected to assign the reference VQ levels may be somehow subjective; nevertheless, given the clear artefacts present in ccECG, this approach can be considered as a good approximation that allows for the evaluation of the automatic SQIs presented in this work; this gives additional information that would not be obtained by only having a reference quality based on beat matching, as it is sometimes done in this type of experiments [8]. Our VQ reference levels allow not only to identify a reference for the quality of the signal in terms of beat matching, but also in terms of the “cleanness” of the complete ccECG morphology, which is a required parameter to be able to compare the selection and classification performance of SQIs.

Besides the evaluation of the usefulness of ccECG signals towards the extraction of features used in sleep apnea detection, the collected data and VQ quality metrics allowed to verify that for a sleep environment, high percentage of the ccECG signals can be found within the highest quality levels: From a set of every best 30-s segment per measurement, 100% of the back signals have a VQ of level 3 of higher, and 86.66% of level 4 or higher, while these percentages are 83.33% and 63.33% respectively for the signals from the side. In addition, the 13.33% of segments from the side located in a VQ level of 1 correspond to problems in misalignment with the sensors, which indicates that the percentage of good quality signals is likely to increase when using a system with a higher-density electrode array providing more combinations to select from. Although signals of subjects lying in their stomach were not recorded due to a standard electrode separation in the setup; ECG from this position is expected to have similar quality, given that a denser array with shorter electrode separation is provided, which would allow for a selection from a higher number of possible configurations, and is likely to solve the problems recording signals from the side that was identified for some of the volunteers.

It is worth noting that the acquired signals did not include transition between different sleeping positions, which is an important source of artefacts and noise; the SQIs presented are expected to identify these artefacts, but also to detect an optimal configuration for the new sleeping position. In addition, the applicability of the method for general population, and specially for sleep apnea patients, needs to be further validated by a study that involves people with this sleep condition being simultaneously monitored by the Polysomnography gold standard method. Future work will include this type of evaluation during a complete night at a sleep laboratory, which will allow to evaluate the performance regarding identification of sleep apnea epochs, as well as the characteristics of the signal for the transition between different sleeping positions, and to identify if there are any differences when the subjects are really sleeping and not in a simulated sleep environment.

## 5. Conclusions

This study presented, for the first time, the evaluation of a multi-channel ccECG system for the extraction of ECG features previously demonstrated to be highly effective in the automatic detection of sleep apnea. In addition, it evaluated multiple SQIs for the automatic classification of the useful signals and the selection of the best available channel, and demonstrated the reliability of ccECG signals acquired from a sleep environment. Results show that HR and HRV related features can accurately be extracted from ccECG signals (Kruskal-Wallis test *p*-values of 0.94 and 0.98), when appropriate higher-quality selection is previously performed. In this sense, two of the presented SQIs (corrSQI and bSQI) had a high performance when classifying usable signals, with accuracies between 86% and 91%; and corrSQI allowed to perform a best-channel selection with an accuracy of up to 92%. This demonstrates not only the suitability of ccECG for unobtrusive apnea detection, but also the usefulness of these SQIs as a pre-processing step. Extracted ccECG morphology-related features did not present enough similarity to the reference ECG counterpart, which is why future work will involve apnea patients, and an evaluation of the features to be used for ccECG. Besides the high reliability of HR and HRV related feature extraction, and the demonstration of SQIs as an indispensable tool for ccECG signal selection and classification, results also evidence a high signal coverage across subjects, with 100% of the best-segments per measurement having a signal quality with identifiable ECG R peaks when recorded from the back, and 83.33% when recorded from the side. Since respiration is an important signal in sleep apnea, an additional method of obtaining respiratory activity in an unobtrusive way (different to EDR) would be of great benefit when combined with the ccECG measurements for the detection of sleep apnea.

## Figures and Tables

**Table 1:** Spectral ranges for the 6 SDR calculated quality indicators.

Parameter	[a–b] (Hz)	[c–d] (Hz)
SDR1	[5–14]	[5–50]
SDR2	[5–14]	[0–50]
SDR3	[5–20]	[0–50]
SDR4	[5–30]	[0–50]
SDR5	[8–20]	[0–50]
SDR6	[3–20]	[0–50]

**Table 2:** Conditions for narrowing down initial channel pre-selection.

Parameter	Condition
HR	35 bpm < HR < 165 bpm
HRV	90 ms < HRV < 600 ms
Peak-to-peak value (Vpp)	0.1 mV< Vpp < 3 mV
RMS value (Vrms)	0.005 mV< Vrms <0.7 mV

Average window values were compared against conditions. Limits were selected based on normal physiological ranges, taking in to account the possible reduction in Vpp and Vrms due to capacitive coupling.

**Table 3:** Visually identified non-contact ECG quality for the collection of every best segment per measurement.

Subject Position in Mattress	Visual Quality Levels ^1^					
	# 1	# 2	# 3	# 4	# 5	# 6
Lying on back	0.00%	0.00%	13.33%	33.33%	40.00%	13.33%
Lying on right/left side	13.33%	3.33%	20.00%	40.00%	13.33%	10.00%

^1^ Tables Visual quality levels defined as in Figure 2. Presented values were obtained when including the best 30-s segment from each 10-min measurement.

**Table 4:** Summary of beat detection and heart rate statistics of non-contact ECG measurements compared with the reference when including the best channel from each measurement.

	Metric	Sensitivity	Positive Predictive Value	Average of Tachogram Correlations
Position				
All positions		95.02%	96.09%	96.94%
Lying on back		98.84%	98.83%	99.18%
Lying on right/left side		92.46%	94.23%	95.60%

**Table 5:** Test results for segment classification into usable or not usable ccECG considering level 3 as threshold for usable signal.

	Lying on Back, Level 3 as Threshold					Lying on Side, Level 3 as Threshold					All Segments, Level 3 as Threshold				
	TH	Sens	Spec	PPV	Acc	TH	Sens	Spec	PPV	Acc	TH	Sens	Spec	PPV	Acc
**DR1**	0.68	0.64	0.12	0.20	0.25	0.43	0.56	0.94	0.90	0.76	0.44	0.55	0.94	0.86	0.78
**SDR2**	0.08	0.42	0.81	0.43	0.72	0.00	1.00	0.00	0.49	0.49	0.08	0.40	0.72	0.49	0.59
**SDR3**	0.19	0.44	0.68	0.32	0.62	0.00	1.00	0.00	0.49	0.49	0.09	0.39	0.75	0.51	0.60
**SDR4**	0.23	0.56	0.33	0.22	0.39	0.46	0.47	0.85	0.75	0.66	0.00	1.00	0.00	0.40	0.40
**SDR5**	0.08	0.44	0.78	0.40	0.69	0.00	1.00	0.00	0.49	0.49	0.05	0.60	0.24	0.35	0.39
**SDR6**	0.28	0.47	0.72	0.36	0.66	0.00	1.00	0.00	0.49	0.49	0.24	0.45	0.64	0.45	0.56
**Kurtosis**	3.98	0.68	0.88	0.66	0.83	0.00	1.00	0.00	0.49	0.49	0.00	0.00	1.00	NaN	0.60
**KurtosisMod**	3.87	0.79	0.74	0.50	0.75	4.35	0.75	0.60	0.64	0.68	4.35	0.77	0.64	0.59	0.69
**bSQI**	0.63	0.97	0.71	0.53	0.77	0.72	0.86	0.54	0.64	0.70	0.72	0.89	0.60	0.60	0.72
**corrSQI**	0.43	**0.89**	**0.92**	**0.78**	**0.91**	0.47	**0.84**	**0.71**	**0.74**	**0.77**	0.47	**0.85**	**0.79**	**0.73**	**0.82**

TH = Threshold; Sens: Sensitivity; Spec: Specificity; PPV: Positive Predictive Value; Acc: Accuracy. Usable signals: VQ 3 to VQ 6.

**Table 6:** Test results for segment classification into usable or not usable ccECG considering level 4 as threshold for usable signal.

SQI Name	Lying on Back, Level 4 as Threshold					Lying on Side, Level 4 as Threshold					All Segments, Level 4 as Threshold				
	TH	Sens	Spec	PPV	Acc	TH	Sens	Spec	PPV	Acc	TH	Sens	Spec	PPV	Acc
**SDR1**	0.63	0.48	0.78	0.69	0.63	0.59	0.59	0.21	0.71	0.50	0.60	0.57	0.27	0.61	0.47
**SDR2**	0.17	0.65	0.77	0.74	0.71	0.12	0.57	0.79	0.90	0.62	0.13	0.59	0.81	0.87	0.67
**SDR3**	0.22	0.66	0.81	0.78	0.73	0.18	0.59	0.76	0.89	0.63	0.20	0.62	0.79	0.86	0.68
**SDR4**	0.27	0.66	0.80	0.78	0.73	0.22	0.58	0.78	0.90	0.62	0.23	0.58	0.83	0.87	0.67
**SDR5**	0.12	0.62	0.91	0.88	0.76	0.12	0.59	0.78	0.90	0.64	0.12	0.60	0.85	0.89	0.68
**SDR6**	0.36	0.66	0.80	0.77	0.73	0.28	0.60	0.77	0.89	0.64	0.32	0.64	0.78	0.85	0.68
**Kurtosis**	7.21	0.74	0.74	0.75	0.74	0.00	0.00	1.00	NaN	0.23	0.00	0.00	1.00	NaN	0.33
**KurtosisMod**	4.67	0.71	0.75	0.75	0.73	4.62	0.71	0.74	0.90	0.71	4.67	0.71	0.74	0.85	0.72
**bSQI**	0.90	**0.90**	**0.84**	**0.85**	**0.87**	0.79	**0.88**	**0.83**	**0.95**	**0.87**	0.82	**0.89**	**0.85**	**0.92**	**0.87**
**corrSQI**	0.70	**0.85**	**0.89**	**0.89**	**0.87**	0.57	**0.85**	**0.86**	**0.95**	**0.86**	0.58	**0.84**	**0.90**	**0.94**	**0.86**

TH = Threshold; Sens: Sensitivity; Spec: Specificity; PPV: Positive Predictive Value; Acc: Accuracy. Usable signals: VQ 4 to VQ 6.

**Table 7:** Confusion matrix for *corrSQI* quality indicator used to classify the non-contact ECG segments in to usable, limited usability and not usable.

	Usable	Limited Usability	Not Usable	Class Sens.	Total Class Segments
Usable	273 (28.92%)	30 (3.18%)	10 (1.06%)	**0.87**	313
Limited Usability	76 (8.05%)	78 (8.26%)	100 (10.59%)	0.31	254
Not Usable	11 (1.17%)	56 (5.93%)	310 (32.84)	**0.82**	377

Presented data corresponds to the classification performance in the test set including all measurements (from back and side), and applying thresholds of 0.45 and 0.56 obtained by a LDA in the training set. Overall multi-class accuracy of 70% was obtained.

**Table 8:** Test results for best-channel non-contact ECG selection using signal quality indicators.

	Lying on Back				Lying on Side				All Segments			
	Sens0	PPV0	Sens1	PPV1	Sens0	PPV0	Sens1	PPV1	Sens0	PPV0	Sens1	PPV1
**SDR1**	0.31	0.30	0.36	0.35	0.26	0.24	0.52	0.48	0.28	0.26	0.46	0.43
**SDR2**	0.50	0.49	0.56	0.55	0.34	0.31	0.42	0.39	0.40	0.38	0.48	0.45
**SDR3**	0.47	0.45	0.53	0.52	0.30	0.27	0.36	0.33	0.37	0.34	0.43	0.40
**SDR4**	0.47	0.45	0.51	0.49	0.26	0.24	0.34	0.31	0.34	0.32	0.41	0.38
**SDR5**	0.51	0.49	0.53	0.52	0.32	0.29	0.39	0.35	0.39	0.37	0.44	0.42
**SDR6**	0.51	0.50	0.58	0.57	0.30	0.28	0.37	0.34	0.39	0.36	0.45	0.42
**Kurtosis**	0.61	0.60	0.73	0.72	0.57	0.52	0.70	0.64	0.59	0.55	0.72	0.67
**KurtosisMod**	0.66	0.65	0.82	0.80	0.57	0.52	0.70	0.63	0.61	0.57	0.74	0.70
**bSQI**	0.73	0.71	0.86	0.84	0.60	0.55	0.66	0.60	0.65	0.61	0.74	0.69
**corrSQI**	**0.89**	**0.87**	**0.94**	**0.92**	**0.78**	**0.71**	**0.84**	**0.77**	**0.82**	**0.77**	**0.88**	**0.82**
**SQI-Fusion**	0.82	0.80	0.90	0.88	0.63	0.57	0.71	0.65	0.70	0.66	0.79	0.74
**SQI-Fusion *w*/*o* corrSQI**	0.75	0.73	0.85	0.83	0.59	0.54	0.67	0.61	0.65	0.61	0.74	0.69

Sens0 & PPV0: Sensitivity and Positive Predictive Value for best-channel selection without any error; Sens1 & PPV1: Sensitivity and Positive Predictive Value for best-channel selection when considering the selection of second-best channel as a match, if the channel is usable (Visual Quality Higher than level 2).
